# Enhancing assertive community treatment with cognitive behavioral social skills training for schizophrenia: study protocol for a randomized controlled trial

**DOI:** 10.1186/s13063-015-0967-8

**Published:** 2015-09-30

**Authors:** Eric Granholm, Jason L. Holden, David Sommerfeld, Christine Rufener, Dimitri Perivoliotis, Kim Mueser, Gregory A. Aarons

**Affiliations:** Veterans Affairs San Diego Healthcare System (116B), 3350 La Jolla Village Drive, San Diego, CA 92161 USA; Department of Psychiatry, University of California, San Diego, CA USA; Boston University, Boston, MA USA

**Keywords:** Schizophrenia, Functioning, Social skills, Cognitive behavior therapy, Assertive community treatment, Implementation, Evidence-based practice, Community practice, Concept mapping

## Abstract

**Background:**

Schizophrenia leads to profound disability in everyday functioning (e.g., difficulty finding and maintaining employment, housing, and personal relationships). Medications can effectively reduce positive symptoms (e.g., hallucinations and delusions), but they do not meaningfully improve daily life functioning. Psychosocial evidence-based practices (EBPs) improve functioning, but these EBPs are not available to most people with schizophrenia. The field must close the research and service delivery gap by adapting EBPs for schizophrenia to facilitate widespread implementation in community settings. Our hybrid effectiveness and implementation study represents an initiative to bridge this divide. In this study we will test whether an existing EBP (i.e., Cognitive Behavioral Social Skills Training (CBSST)) modified to work in practice settings (i.e., Assertive Community Treatment (ACT) teams) commonly available to persons with schizophrenia results in better consumer outcomes. We will also identify key factors relevant to developing future CBSST implementation strategies.

**Methods/Design:**

For the effectiveness study component, persons with schizophrenia will be recruited from existing publicly funded ACT teams operating in community settings. Participants will be randomized to one of the 2 treatments (ACT alone or ACT + Adapted CBSST) and followed longitudinally for 18 months with assessments every 18 weeks after baseline (5 in total). The primary outcome domain is psychosocial functioning (e.g., everyday living skills and activities related to employment, education, and housing) as measured by self-report, testing, and observation. Additional outcome domains of interest include mediators of change in functioning, symptoms, and quality of services. Primary analyses will be conducted using linear mixed-effects models for continuous data. The implementation study component consists of a structured, mixed qualitative-quantitative methodology (i.e., Concept Mapping) to characterize and assess the implementation experience from multiple stakeholder perspectives in order to inform future implementation initiatives.

**Discussion:**

Adapting CBSST to fit into the ACT service delivery context found throughout the United States creates an opportunity to substantially increase the number of persons with schizophrenia who could have access to and benefit from EBPs. As part of the implementation learning process training materials and treatment workbooks have been revised to promote easier use of CBSST in the context of brief community-based ACT visits.

**Trial registration:**

ClinicalTrials.gov NCT02254733. Date of registration: 25 April 2014.

## Background

In the United States, schizophrenia affects more than 3 million residents, costs more than US$62 billion annually (US$30 billion in direct healthcare), and leads to profound disability in everyday functioning (e.g., difficulty finding and maintaining employment, housing, and personal relationships). Medications have been shown to be effective at reducing positive symptoms (e.g., hallucinations and delusions), but they do not improve daily life functioning. Psychosocial evidence-based practices (EBPs) that improve functioning have been developed, tested and recommended in best practice guidelines, but these EBPs are rarely available to most people with schizophrenia [1]. The gap between research and service delivery must be closed by adapting EBPs for schizophrenia to facilitate widespread implementation in community settings. This hybrid effectiveness and implementation [2] study protocol represents an initiative to bridge this divide by testing whether an existing EBP, called cognitive behavioral social skills training (CBSST), which has been modified to work in the context of Assertive Community Treatment (ACT) teams, commonly available to persons with schizophrenia in public sector community practice settings, results in better consumer outcomes. In addition, Concept Mapping (CM) will be utilized to facilitate the identification of factors relevant to inform the development of future implementation strategies.

### Interventions targeting functioning in schizophrenia

**Fig. 1:**
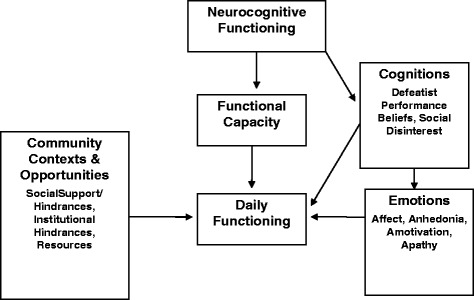
Model of functional outcome in schizophrenia

Interventions guided by research on factors that contribute to functional impairment in schizophrenia are needed. In this section, we briefly describe a model of functional outcome in schizophrenia (see Fig. 1) that includes the determinants of functional impairment targeted by CBSST, including skill competence and defeatist performance attitudes. It is well established that neurocognitive deficits are associated with functional impairment in schizophrenia [3, 4], but the relationship between neurocognitive impairment and real-world outcome in schizophrenia is mediated by several factors, such as functional skill capacity or the ability to perform the skills needed to function in the community (e.g., grocery shopping, writing checks to pay bills, using an automated teller machine) [5–8]. Functional skills like interpersonal communication skills can be systematically trained using social skills training (SST), which is one key element of the CBSST program.

Some consumers with adequate functional skill capacity, however, still have poor community functioning. Other factors influence whether skills are performed in daily life settings, including environmental factors (e.g., access to normative community settings, social support) and personal factors (e.g., beliefs/expectations, self-efficacy, motivation, mood) [9–12]. In particular, the premise that inaccurate beliefs and expectations about the likely consequences of performing a behavior are a major contributor to real-world functioning is a key component of the cognitive model that guides cognitive behavior therapy (CBT) interventions for schizophrenia [11]. Grant and Beck [13] reported that dysfunctional performance beliefs (e.g., “I always fail so why try?”) are associated with negative symptoms and poor real-world functioning in schizophrenia. They have suggested that neurocognitive impairments, stigma, and other illness-related factors, can lead to discouraging everyday failure experiences that lead to overgeneralized negative expectancies and defeatist beliefs. These defeatist beliefs can be modified using CBT, which is another key component of the CBSST program.

The intervention delivered in this study, therefore, integrates EBPs that target several factors in this functional outcome model of schizophrenia. CBSST uses SST to target functional skill capacity and CBT to target cognitions that interfere with effective skill performance in the real world. In addition, the ACT staff who deliver CBSST also help clients take advantage of community and institutional supports that promote functioning.

SST and CBT are well-validated EBPs that have been shown to improve functioning and are recommended in several treatment guidelines for schizophrenia [14–16]. In a meta-analysis of 35 clinical trials of CBT for schizophrenia [14], the majority of studies focused on positive symptoms as primary treatment targets, but CBT had beneficial impact on functioning outcomes with moderate to large sizes (*d* = 0.378) comparable to that for positive symptoms (*d* = 0.372). Additionally, numerous studies of consumers with schizophrenia have shown that SST improves psychosocial functioning (*d* = 0.52) [15, 16].

Our prior research has demonstrated that CBSST, as a bundling of these two potent interventions, effectively improves functioning in schizophrenia [17, 18]. In an initial randomized clinical trial [17, 18], comparing CBSST with Treatment as Usual (TAU), consumers with schizophrenia in CBSST showed significantly better functioning on the Independent Living Skills Survey (ILSS), a self-report measure of everyday functional living skills for consumers with severe mental illness [19]. This difference was maintained at 1-year follow-up [18]. Two subsequent comparative effectiveness research randomized clinical trials compared CBSST with an active goal-focused supportive contact condition (GFSC) in consumers with schizophrenia and both trials found significantly greater self-reported community functioning on the ILSS in CBSST relative to GFSC [20, 21]. In addition, one trial also found significantly greater improvement in amotivation/asociality negative symptoms in CBSST relative to GFSC [20]. Finally, these clinical trials also found evidence that improvement in defeatist performance beliefs was an important mechanism of change in CBSST, because defeatist beliefs improved significantly more in CBSST relative to GFSC and greater improvement in defeatist attitudes was associated with greater improvement in functioning [20, 21]. These results indicate that CBSST can effectively improve functioning in persons with schizophrenia in a research clinic setting when delivered by expert highly-educated providers, but CBSST has not been evaluated in the context of a public sector community mental health setting when delivered by frontline clinicians. It is rare to find such services provided in the community.

ACT is one of the few EBPs for schizophrenia that is widely implemented in community mental health programs throughout the United States. ACT is better characterized as a different way of organizing and tailoring the delivery mental health services to a particular special population of consumers who do not typically access these services on their own (or only through emergency services), rather than a specific treatment or intervention like SST or CBT. ACT is a team treatment approach with shared, low caseloads, community-based service delivery, and a focus on reducing hospitalizations, maintaining housing, and improving daily living skills; however, ACT has little impact on psychosocial functioning [22–24]. Because ACT is widely utilized, the ACT team provides a promising platform to achieve broader implementation of recovery-oriented EBPs like CBSST in community mental health programs.

ACT teams provide an optimal platform for an adapted version of CBSST for several additional reasons: 1) ACT staff have a lower caseload than other forms of case management, so they typically provide weekly visits and have more time per visit to spend in psychotherapy, 2) lower functioning consumers who are most in need of interventions to improve their functioning are typically assigned to ACT teams, so enhancing ACT by adding CBSST may improve the impact of ACT on psychosocial functioning for consumers who need this the most, 3) ACT staff have some education in mental health (e.g., typically bachelor level) and experience interacting with consumers, and already have rapport with consumers on their teams. Recognizing the potential for utilizing ACT teams as a mechanism for substantially increasing the utilization of EBPs to address functioning in consumers with schizophrenia, we adapted CBSST to be delivered by ACT teams (adaptations are described below).

We conducted a feasibility trial of an abbreviated version of our manualized ACT-adapted, team-delivered CBSST that was provided to a small sample of persons with schizophrenia or schizoaffective disorder (*N* = 16) [25]. This feasibility study was conducted using sixteen clinicians from two ACT teams in the San Diego County Mental Health System. Typical of ACT, staff members had bachelor’s degrees (most commonly) or master’s degrees and 3–5 years of experience working as mental healthcare providers. The ACT-adapted CBSST intervention was generally delivered with adequate fidelity, despite limited training and supervision. Further modifications were incorporated into the ACT-adapted CBSST intervention (e.g., enhanced training) to promote successful, high-fidelity implementation in community settings.

The hybrid effectiveness and implementation study described below takes the next logical step by examining the effectiveness of, and implementation considerations for, CBSST adapted for real-world community ACT settings. By training existing staff in community mental health system ACT teams to deliver CBSST, we will evaluate whether the adapted CBSST will improve psychosocial functioning outcomes in people with schizophrenia in the context of a real-world mental health delivery system. Improved psychosocial functioning in consumers served by ACT could potentially accelerate the progress of these consumers, allowing for more rapid graduation from ACT teams to less intensive services, thereby increasing access to ACT services for other consumers or resulting in cost savings in ACT services required to meet consumer needs. While information on direct costs of intervention delivery will not be available, this study will inform aspects of provider time and burden (e.g., training and supervision time) that would be related to cost, as well as provider impressions of added burden and cost in the implementation study component.

Consistent with a “Hybrid Type 1” design, the primary aims of the proposed project are to evaluate whether the CBSST program, when provided by ACT staff in the context of usual ACT services, improves functioning in schizophrenia more than ACT alone. A secondary aim is to examine potential mechanisms of change in functioning in CBSST, which could have implications for refining the CBSST treatment program. Specifically, CBSST attempts to teach social skills and modify defeatist performance beliefs (e.g., “It’s not worth the effort,” “It won’t be fun”) that can interfere with community functioning [13], so we will examine whether improvement in social skill competence and reduction in defeatist performance beliefs mediate improvements in psychosocial functioning in CBSST. Finally, an exploratory aim is to use mixed qualitative-quantitative methods (i.e., CM, qualitative interviews, staff and supervisor surveys) to identify barriers to, and facilitators of, successful implementation of CBSST across ACT teams. We will examine the perspectives of multiple stakeholders in the service system, including administrators and clinicians in provider organizations, and consumers. We expect that there will be both congruence and differences in perceptions of barriers/facilitators across stakeholder groups and that policy, funding, organizational process, and provider and consumer preferences will be important in implementation success and generalizability.

### Specific aims

#### Aim 1-effectiveness

To examine whether adding CBSST to ACT improves rehabilitation outcomes compared to ACT alone in consumers with schizophrenia.

#### Aim 2-effectiveness mediators

To determine whether improvement in social competence and reduction in defeatist beliefs mediate changes in psychosocial functioning in CBSST in consumers with schizophrenia.

#### Aim 3-implementation barriers and facilitators

To use mixed-methods with multiple stakeholder groups to identify barriers to, and facilitators of, successful implementation of CBSST on ACT teams that can inform future wider dissemination.

If effectiveness of CBSST in the hands of community providers is demonstrated, this project could have significant impact on dissemination and implementation of EBPs for severe mental illness on ACT teams in the United States. As a hybrid effectiveness and implementation study, the project would also identify barriers to, and facilitators of, successful implementation of EBPs on ACT teams to inform wider dissemination of EBPs in public mental health systems across the United States. From a public health standpoint, broader dissemination of such enhanced ACT interventions that improve psychosocial functioning in schizophrenia could have significant personal, societal, and economic benefit.

## Methods

The design of this hybrid effectiveness and implementation study is consistent with the “Hybrid Type 1” approach [2] in that the effectiveness study component is the primary research emphasis consisting of a randomized controlled trial. The implementation study component is secondary and includes a mixed qualitative-quantitative methodology to characterize and assess the implementation experience from multiple stakeholder perspectives in order to inform future implementation initiatives.

Participants with schizophrenia or schizoaffective disorder will be randomized to one of the 2 treatments (ACT alone or ACT + CBSST) and followed longitudinally for 18 months. All participants will continue to receive pharmacotherapy and other services they receive in standard ACT care. All participants will provide written informed consent after a study staff member provides a detailed description of the procedures, risks, and benefits. Participants are encouraged to ask any questions they may have about the study or study procedures prior to signing the consent form. It will be made clear to participants that their participation in the study is completely voluntary and declining to participate will not in any way impact the services or benefits to which they are entitled. A participant’s understanding of key study components, including specific details regarding randomization, benefits/risks, their role in the study, and the voluntary nature of research participation will be queried through a series of questions. If a participant cannot correctly answer all of these questions after the study is explained three times, then the participant will not be enrolled.

This study is funded by the USA National Institute of Mental Health (NIMH), and was reviewed and approved for the ethical treatment of human subjects by the Institutional Review Board of the Department of Veterans Affairs San Diego Healthcare System and the University of California, San Diego. Ethical review and approval was also provided by the Research Committee of the San Diego County Mental Health System.

We will describe the effectiveness portion of the hybrid study design first followed by a discussion of the implementation study design.

### Effectiveness study design (Aim 1 and Aim 2)

This is a randomized clinical trial comparing 2 treatment conditions: ACT alone, and ACT + CBSST. We will train staff (*N* = 90) from 7 ACT teams to deliver individual CBSST to consumers with schizophrenia or schizoaffective disorder (*N* = 176; based on power analysis). Consumers will be randomized within ACT teams to one of the treatment conditions and followed for 18 months, with assessments conducted every 18 weeks after baseline (5 in total). A 1-day training workshop will be provided to ACT staff who will then begin to provide CBSST to consumers randomized to ACT + CBSST, while receiving 30 minutes of weekly group consultation/training from experienced research clinicians.

### Participant eligibility

To maximize generalizability, the following minimal inclusion criteria will be used: 1) voluntary informed consent, 2) age 18 or older, 3) *Diagnostic and Statistical Manual of Mental Disorders, 4th edition, text revision* (DSM-IV-TR) diagnosis [26] of schizophrenia or schizoaffective disorder, 4) receiving ACT services for at least 3 months, 5) no prior SST or CBT in the past 3 years, and 6) living in the community for at least the past month. There were no other inclusion/exclusion criteria. To confirm the diagnosis of schizophrenia or schizoaffective disorder we will use the Structured Clinical Interview for DSM-IV (SCID) to collect diagnostic data, followed by a consensus diagnosis with an experienced diagnostician procedure that utilizes all available records and the structured interview. We will also obtain demographic data, medical and pharmacologic history about pertinent medical illnesses to help inform diagnosis.

### Randomization procedures and rationale

Randomization of consumers to treatment groups will be stratified by ACT team and gender within ACT team. Stratification by gender will be conducted because women with schizophrenia tend to have better social competence and social/community functioning, and fewer hospitalizations than men [27–32]. An independent statistician will create randomization sequences and assign cases to conditions.

We considered randomizing ACT teams to conditions, rather than consumers within teams, in order to guard against possible “bleed” of the CBSST intervention into the ACT-only condition, but opted against this for two reasons. First, possible contamination across treatment conditions is prevented by using the standardized treatment manual, as well as through provider supervision strategies. The use of standardized manuals in psychotherapy research minimizes the likelihood of contamination of treatments from one condition to another. During weekly supervision, study consultants facilitate detailed discussions about intervention delivery, provide feedback based on recordings of both ACT-only, and ACT + CBSST sessions to improve CBSST skills, as well as cautioning providers about teaching CBSST skills in ACT-only sessions, if recordings indicate any intervention bleed.

Second, the number of ACT teams required to have sufficient power to test our main hypotheses in a cluster randomized controlled trial is large and would not permit the same rigorous evaluation of outcomes critical to testing the CBSST model. We were concerned that the number of expected ACT teams (7) would not provide a sufficient unit-level sample size for randomization by teams to result in matched groups at baseline. In addition, a complex stratification according to multiple consumer characteristics at baseline would be difficult to accomplish with the proposed sample size and study timeline.

All consumers in both conditions will continue to have access to pharmacological treatments and all other ACT services, with no study-related restrictions. We will calculate total daily dose of antipsychotic medications at each assessment as chlorpromazine equivalents (mg/day CPZE). At each assessment, antipsychotic medication type (typical versus atypical) and starting and stopping of other psychotropic medications (antidepressants, mood stabilizers) will be recorded.

### ACT alone/usual care condition

ACT teams in the San Diego County Mental Health System typically consist of case managers/care coordinators, substance abuse specialists, some nurse specialists, and a psychiatrist. The education level of ACT staff is varied, but staff typically have a bachelor’s or master’s degree, with a few staff having MDs (psychiatrists), RN/LVN degrees or substance abuse treatment certification. ACT team members provide a combination of services, including case management, round-the-clock crisis intervention, acting as a payee, interacting with collaterals, monitoring clients, responding to crisis, and care coordination. ACT treatment, including the proposed CBSST-enhanced ACT, is provided in the community (typically not in a clinic office).

### CBSST condition

Consumers in both ACT-only, and ACT + CBSST will receive ACT visits of standard frequency and duration from the same providers. ACT teams attempt to visit consumers at least once a week in both conditions. More frequent visits may be provided if consumers are in crisis; less frequent if consumers are not available when staff attempt a visit. The individual ACT + CBSST intervention is designed to be conducted in the context of standard visits (structured intervention time replaces supportive case management contact time). Providers are informed that the study goal is to deliver at least 36 sessions of CBSST to consumers in ACT + CBSST during these regular ACT visits over the course of the 18-month protocol. The CBSST intervention is delivered in the context of these regular ACT visits, without adding additional meetings with ACT staff to deliver CBSST. Thus, the two conditions should be matched for amount of attention and care.

Cognitive Behavioral Social Skills Training (CBSST) [17, 33, 34] integrates CBT [35–38] and SST [39, 40] techniques. The treatment manual includes a consumer workbook that describes the skills and includes homework assignment forms. Cognitive therapy is combined with role-play practice of communication skills and problem-solving training. The proposed ACT-adapted, team-delivered individual CBSST intervention will be delivered in three 6-session modules described below for a total of 18 individual therapy sessions, with participants expected to complete the sequence of 3 modules twice, for a total of 36 sessions. Repeating the modules in this way provides skills practice consistent with behavioral learning principles that can facilitate learning in spite of cognitive impairment. Repetition may also improve sense of mastery and self-efficacy. This is not simply rote repetition. Skills are applied to different thoughts, problems, and social situations arising in the lives of consumers over the course of the program. If one CBSST session is completed each week, full exposure to all skill content could be completed within 9 months, but ACT staff will be permitted to use CBSST interventions for consumers randomized to CBSST throughout the 18-month protocol. ACT staff will be asked to complete a form documenting whether CBSST interventions and/or other ACT services were delivered during standard team encounters with consumers to track the number of sessions with CBSST, and will be asked to obtain audio recordings of as many sessions as possible for fidelity ratings. After delivering all the CBSST content, therapists will be free to select any of the material in the manual to personalize the sessions to the individual needs of the client. Delivering booster sessions during follow-up is common in CBT trials, and the community-based nature of ACT provides ideal opportunities for staff members to prompt consumers to use CBSST skills in appropriate situations, either during the course of providing CBSST sessions or after completion of the modules. Thus, the present study is not aimed at evaluating the durability of CBSST following cessation of treatment, which has already been amply demonstrated in multiple studies of CBT [15, 41] and SST [16, 17], and in our previous work with CBSST [14, 16], but rather to examine whether adding CBSST to ACT improves the overall impact of ACT on functioning compared to ACT alone.

ACT staff will provide CBSST in the context of their regular ACT visits. Sessions are designed to be approximately 30 minutes long, but include core content that can be covered in less time. As with all other ACT services, CBSST will be provided in the community at sites chosen by staff and consumers (e.g., residential settings, clubhouses, coffee shops, parks). The ACT model is a team treatment model, so consumers will likely have different clinicians deliver different sessions, depending on which ACT team member visits the consumer that week. Continuity of treatment (e.g., sharing information between clinicians about homework assignments and session progress) will be managed during the standard ACT morning meetings.

#### CBSST cognitive skills module

Cognitive therapy is the exclusive focus of this module but these techniques are also used throughout the other two modules. Importantly, the cognitive therapy approach focuses primarily on teaching cognitively-based coping skills for addressing obstacles to goals and upsetting feelings, rather than being based on complex formulations or schema-based work. These interventions involve practicing simple steps for modifying problematic beliefs that are compatible with other skills training approaches like SST. Less training is required for novice clinicians typical of ACT staff to become proficient at these cognitive therapy skills, compared to more complex schema or formulation-based CBT approaches. The manualized CBSST intervention is a standardized curriculum that can be easily delivered by any clinician on the ACT team.

Cognitive interventions are used to address symptoms and challenge defeatist or other maladaptive beliefs that interfere with the pursuit of goals or use of skills in real-world situations, such as negative expectancies (“It won’t be fun”), beliefs about lack of self-efficacy (“I can’t do it”), and delusions (“Spirits will harm me”). By challenging defeatist performance beliefs, consumers are more likely to engage in functional behaviors and use the skills they have. Consumers are introduced to the general concepts of CBT, including the relationship between thoughts, actions and feelings (generic cognitive model), automatic thoughts, thought challenging by examining evidence for beliefs, and mistakes in thinking (e.g., jumping to conclusions, fortune telling, all-or-none thinking). Through discussion, thought records, and homework assignments, consumers are taught to identify thoughts, examine relationships between thoughts, feelings and behaviors, and correct mistakes in thinking. Behavioral experiments are conducted inside and outside therapy sessions (homework), in order to gather evidence to evaluate beliefs. To simplify learning and help consumers remember and use cognitive techniques in everyday life, mnemonic aids are provided (e.g., laminated cards describing skills). For example, for thought challenging, we use an acronym, “The 3C’s: Catch it, Check it, Change it.” The “it” refers to a thought. Consumers are taught to use the 3C’s when they are experiencing unpleasant emotions or behaving in a way that interferes with goals (e.g., isolating, not filling out a job application, giving up on a class).

#### CBSST social skills module

The primary goal of this module is to improve communication skills and psychosocial interactions (e.g., how to ask someone for support). The predominant therapeutic technique is the use of behavioral rehearsal in role plays followed by positive and corrective feedback to shape more effective behavioral repertoires. An important focus of role plays is on interacting with roommates, friends and family, making new friends, and effectively interacting with case managers, other service providers and support persons. Expressing pleasant and unpleasant feelings, and making positive requests are emphasized to improve assertive, clear, and comfortable sharing of feelings in social interactions. Improving everyday activities and functioning are common role-play topics for these skills (e.g., asking a roommate to change their behavior; asking someone to go to the movies; assertive interactions with co-workers/ employers). Self-efficacy and performance beliefs are elicited and scaling is used before and after role plays (0–10 ratings of how successful you think you will be/were) to challenge defeatist beliefs about skills.

#### CBSST problem-solving module

As is common in SST and CBT interventions, basic problem-solving skills are taught. CBSST uses the acronym, SCALE – *S*pecify the problem, *C*onsider all possible solutions, *A*ssess the best solution, *L*ay out a plan, and *E*xecute and evaluate the outcome. The focus of this module is on developing plans to solve real-world problems and to establish the steps for achieving goals such as improving one’s living situation or finances, gaining access to transportation, finding a volunteer/paid job, enrolling in classes, increasing leisure activities, and improving relationships. Problems related to illness and disability are also addressed including, coping with symptoms and stress, remembering to take medications, and improving hygiene/health. Thoughts about the expected success/failure of plans are elicited and evidence for success/failure of attempted plans is reviewed to challenge defeatist beliefs.

### Fidelity monitoring

#### ACT fidelity monitoring

Quality of ACT services will be rated for each team by Dr. Monroe-Devita (consultant) using the Dartmouth Assertive Community Treatment Scale (DACTS) [42]. The DACTS consists of 28 items, each rated on a behaviorally-anchored 5-point scale (1 = not implemented; 5 = fully implemented). The mean score for the total scale will be used as the measure of ACT team fidelity. A DACTS score of > 4.0 is considered moderately high fidelity [42]. We will examine whether ACT fidelity is related to outcomes by including it as a covariate in exploratory analyses, but we do not expect fidelity to be related to outcomes, because we expect all ACT programs to be of comparable fidelity

#### CBSST fidelity monitoring

Audio recordings of therapy sessions will be reviewed and rated for CBT fidelity using the Cognitive Therapy Scale for Psychosis (CTS-Psy) [43] and for SST fidelity using an abbreviated version of the Social Skills Training Fidelity Scale [40]. CTS-Psy ratings will be completed for sessions from all three modules because cognitive therapy interventions are used in all modules, but SST ratings will only be completed for the social skills module. The expert consultants will collect session recordings from providers on the ACT teams they will train and rate fidelity of the sessions on an on-going basis throughout the project. The consultants will meet weekly with ACT teams and provide their ratings to team members as a training tool. To incentivize learning the CBSST skills and delivery of CBSST sessions, we offered providers a certificate stating they were “high-fidelity providers” of CBSST when they delivered 10 consecutive sessions that averaged 30 or greater on the CTS-Psy. After training on the CTS-Psy, we have achieved good inter-rater reliability (intraclass correlation coefficient (ICC) = 0.85) [17].

### Clinician training

The proposed training model has been adapted to the ACT model and workload and will include: 1) an initial 1-day workshop; 2) weekly 30-minute face-to-face skill-focused supervision using coaching and role-play modeling; 3) written feedback of fidelity ratings to individual therapists and review of session audio recordings in supervision; 4) monthly “steering committee” meetings with team leaders to provide technical assistance and support for recruitment, implementation and fidelity.

The workshop will be provided by CBSST experts (EG, KM, JH) and will include review of manuals and homework sheets, videotaped demonstration, and role-played/modeled delivery of CBSST. Half the members of each team will attend the workshop one day and the other half will attend a repeat of the same workshop on another day, so half the team will always be available to manage clinical care. ACT staff and leadership have indicated that a workshop longer than 1 day is not feasible. ACT staff turnover will be addressed by having annual workshops for replacement staff, and all workshop content (slides and video recordings of speakers and role plays) available to new providers online (CBSST.org). New staff will also accompany other ACT staff members to observe and co-lead CBSST sessions.

Based on pilot work, we configured a training model that minimized burden on providers by incorporating training and consultation into one of the existing daily “morning meetings” that are part of the ACT model. Providers on ACT teams usually meet each morning to share information about consumers, review and identify consumers’ progress and needs, and plan for who will meet with which consumers to address particular needs or goals. Once each week, the expert consultant will use 30 minutes of the morning meeting to provide training and consultation. Also, ACT services are team-delivered (i.e., different providers typically provide services to the same consumer), so providers of past CBSST sessions must share information with providers of future sessions (e.g., weekly homework assignments, key thoughts and themes from the last session, consumer reactions/feedback about specific skills, etc.). The daily morning meetings provide an opportunity for different providers to share this information to plan sessions.

### Outcome measures

As shown in Table 1, the primary outcome domain is psychosocial functioning. Additional outcome domains of interest include mediators of change in functioning, symptoms, and quality of services. In the following sections we discuss the key measures used for each domain.

**Table 1:** Summary of dependent variables

Outcome domains	Dependent variables
Psychosocial functioning (Primary domain)	
1. Real-world independent living skills	1. ILSS total (primary measure)
Mediators	
1. Defeatist performance beliefs	1. DPAS total
2. Social competence	2. MASC total effectiveness score
Symptoms	
1. Negative symptoms	1. SANS factors
2. Positive and general psychopathology symptoms	2. BPRS positive symptom factor and total
Quality of services	
1. CBSST fidelity	1. CTS-Psy total and SST fidelity total
2. ACT fidelity	2. DACTS overall average
3. CBSST skill knowledge	3. CMT Total

*Abbreviations: ACT* Assertive Community Treatment*, BPRS* Expanded Brief Psychiatric Rating Scale, *CBSST* Cognitive Behavioral Social Skills Training, *CMT* Comprehensive Modules Test, *CTS-Psy* Cognitive Therapy Scale for Psychosis, *DACTS* Dartmouth Assertive Community Treatment Scale, *DPAS* Defeatist Performance Attitude Scale, *ILSS* Independent Living Skills Survey, *MASC* Maryland Assessment of Social Competence, *SANS* Scale for the Assessment of Negative Symptoms, *SST* social skills training

#### Psychosocial functioning measure

While the various psychosocial functioning measures have their own advantages and disadvantages, we selected the ILSS as the primary outcome measure because it has been the primary outcome measure in our prior CBSST studies that demonstrated the efficacy of CBSST [17, 18]. The ILSS is a 51-item self-report measure of everyday functional living skills that takes approximately 15 minutes to administer and assesses whether or not specific functioning behaviors have been performed over the past month in 10 areas: Personal Hygiene, Appearance and Care of Clothing, Care of Personal Possessions (everyday household chores), Food Preparation, Health Maintenance, Money Management, Transportation, Leisure and Community (including socialization), Job Seeking, and Job Maintenance. Responses to items in each domain are averaged, and a total score is computed as the average of all domain scores. Objective functional milestone indicators will also be collected at each assessment time point, including information on employment, educational activity, psychiatric hospitalizations, and current residential status.

#### Mediator measures

The Maryland Assessment of Social Competence (MASC) will be used in the mediator analyses. The MASC is a structured behavioral role-play assessment that measures the ability to resolve interpersonal problems through conversation [44, 45]. The MASC takes about 15–20 minutes to administer and consists of three 3-minute role-play communication scenarios (1 conversation initiation and 2 assertion), during which the consumer interacts with a live confederate who plays a role (e.g., boss) in a problem-oriented situation (e.g., asking for a work shift change). The measure has three parallel sets of scenarios for multiple administrations. Videotaped role plays are rated by blinded raters on dimensions of verbal content, nonverbal communication behavior, and overall effectiveness, which will be the primary MASC variable. The Defeatist Performance Attitude Scale (DPAS) is a 15-item self-report subscale of the commonly-used 40-item Dysfunctional Attitude Scale (DAS) [46, 47] that was derived from factor analysis. The DPAS indexes generalizes defeatist beliefs about one’s ability to perform tasks and effectiveness of social behaviors (e.g. “If you cannot do something well, there is little point in doing it at all,” “If I fail at my work, then I am a failure as a person”). Providers will document the completion of CBSST sessions, including whether homework was completed and the level of consumer participation in CBSST sessions, which will be explored as potential mediators of treatment outcome.

#### Symptom measures

Negative symptoms for the past 2 weeks will be rated using the Scale for the Assessment of Negative Symptoms (SANS) [48]. Based on factor analytic studies, the SANS ratings will be divided into experiential (Avolition/Apathy, Asociality/Anhedonia) and expressive (Affective Flattening and Alogia) factors [49]. In addition, the Expanded Brief Psychiatric Rating Scale (BPRS) [50] will be administered to index positive and general symptoms over the past 2 weeks.

#### Skill knowledge

The Comprehensive Modules Test (CMT) will be administered to assess mastery of the specific content in the three CBSST modules. The CMT was originally developed at UCLA for use with SST modules [39]. Following this format, similar questions with vignettes were developed to assess mastery of communication (max = 11), problem-solving (max = 11), and thought challenging (max = 11) skills. The CMT total score (max = 33) will be used.

### Blind assessments and inter-rater reliability

Research assistants blind to group membership will perform all assessments. We have systematic procedures in place to counsel consumers not to reveal their treatment assignment to assessors and found the blind was maintained in prior trials [17]. Assessors will receive extensive training using videotaped and practice interviews and will not complete assessments until achieving at least 0.80 inter-rater reliability.

### Analyses

Primary analyses will be based on a linear mixed-effects model for continuous data [51, 52]. We will include and test covariates that can impact functioning as appropriate (e.g., ACT team, gender, DPAS, negative symptoms). Analyses will be performed using HLM 6.06 (SSI Inc., Skokie, IL, USA). We will assess multidimensional outcomes, but selected one primary outcome (ILSS), powered the study based on this outcome, and will determine the effectiveness of the intervention based on this outcome (Aim 1).

### CBSST effectiveness analyses (Aim 1)

In the primary mixed-model analysis, the model will include a random intercept, a fixed effect for the five assessment points, and treatment group (ACT alone versus ACT + CBSST) tested in these models as predictors of the primary outcome measure, ILSS, and the secondary functioning and outcome measures (listed in Table 1). Model diagnostics will be used to determine the suitability of an autoregressive error component and nonlinear effects for assessment time.

### CBSST effectiveness mediators (Aim 2)

The primary mediation analysis will be based on the recommendations of Kraemer et al. [53] using a similar linear mixed-models approach. Two conditions must be met for mediation of the treatment effect: 1) correlation between the mediator and treatment (ACT versus ACT + CBSST group effect on MASC or DPAS), and 2) relationship between the mediator and outcome (MASC or DPAS main effect or group × MASC or group × DPAS interaction on ILSS). A statistically significant effect of treatment group on the outcome is not required, according to Kraemer et al. [54], although this will be tested in Aim 1. First, we will test the effect of treatment group and the group × time interaction on the mediator (MASC or DPAS score across all available assessments). Here we are looking for a statistically significant treatment group × y time interaction. Second, we will test the effects of MASC or DPAS change (slope across available assessments) and the MASC or DPAS change × group interaction on the outcome (ILSS across all available assessments) in the model that includes group and time. Here we are looking for a statistically significant MASC or DPAS change main effect or significant MASC or DPAS change × group interactions.

### Statistical power calculation

The methods described by Diggle et al. [54] and Hedeker et al. [55] were used to calculate power, resulting in a sample size of *N* = 176. We assumed an autoregressive covariance structure, with conservative correlation between sequential assessments set at 0.45 and up to 4 covariates in the model. We powered our primary aim (Aim 1) for our primary outcome measure (ILSS) to detect a small to medium group × y time interaction effect. For the 2 (ACT + CBSST versus ACT alone) by 5 (assessment times) mixed-model analysis evaluating the treatment group effect over the course of the entire study, a sample size of 70 per group (20 % dropout) and *α* = 0.05 2-tailed, we would have minimum power of 0.85 to detect a small to medium (0.35) group × y time interaction effect.

### Mixed-method implementation study design (Aim 3)

Empirical literature suggests that effective dissemination and implementation must take into account the complexity of the service system and multiple levels at which change must occur (i.e., system, organization, individual) in conjunction with the technology or intervention to be disseminated [56–58]. In order to enhance the likelihood of success of future implementations, it is important to determine barriers to, and facilitators of, dissemination and implementation of EBPs, such as CBSST, in real-world service settings such as ACT teams [57, 58]. We will conduct an assessment of such factors using CM methodology to guide data collection and analysis [24, 59, 60]. CM is a mixed qualitative-quantitative method particularly useful with diverse stakeholders that may hold different perspectives and experiences with services [59]. We have used CM to identify barriers to, and facilitators of, EBP implementation in a large youth mental health service system [61] and will apply it here.

There are 6 steps in CM: 1) Preparation – participants are identified, the focus is developed, and a schedule of focus group meetings is set, 2) Generation – stakeholders participate in focus groups and brainstorm statements reflecting possible factors that may facilitate or interfere with the implementation of CBSST in ACT teams, 3) Structuring – each participant sorts the statements into piles based on similarity and then rates each statement on importance and changeability (described below), 4) Representation – data are used to conduct multidimensional scaling wherein each statement is a point on a “Concept Map” with statements piled together by more people closer to each other and cluster analysis is then used to aggregate similar groups of statements into clusters (described below), 5) Interpretation –- the investigators work with stakeholders to reach a consensus for labels and interpretations of the different clusters, and 6) Utilization involves using the maps to help address the original focus, in this case, to develop an implementation intervention to facilitate subsequent CBSST implementation in ACT teams in other settings. Thus, we will use an established method with the appropriate stakeholders to identify barriers to, and facilitators of, implementation and sustainability of CBSST on ACT teams.

The CM steps described above will be used to collect and organize the CM data and illustrate emergent concepts based on study participants’ responses. Initial CM data collection will occur after 7 ACT teams are trained and have delivered CBSST for at least 6 months. Investigators will meet with members from six stakeholder groups: consumers, ACT team members, ACT team leaders, organization administrators, University of California, San Diego (UCSD) research team members, and county representatives. A total of 75–100 participants will complete CM study-related activities. We will explain that the goal is to identify barriers to, and facilitators of, CBSST implementation in ACT teams in public sector mental health settings. The experience of being involved in the implementation of CBSST on these ACT teams will serve as the focal experience. If upper management and policy stakeholders lack in-depth knowledge of the intervention and setting we will provide detailed descriptions and engage the group in discussion to illustrate the process of implementation and service delivery. Thus, collectively, stakeholder participants will be familiar with the intervention, training requirements, intervention duration and frequency, case-manager experience/education, perceived cost/benefit, training experience, and consumer experience with the intervention.

The group will develop a focus statement to guide group brainstorming. An example focus statement is: *“What are the factors that influenced the implementation and use of CBSST in ACT teams in this mental health system?”* Study investigators will then facilitate focus group sessions with each stakeholder group separately in order to promote candid responses and reduce desirability effects while brainstorming barriers to, and facilitators of, implementation and sustained use of CBSST in ACT teams [60]. It is likely that a large number of statements (e.g., 200) will be generated when aggregated across stakeholder groups. Our past experience has shown that by eliminating duplicate statements and combining similar statements, a large number of statements can be distilled into about 100 distinct statements [61]. After randomly renumbering statements to minimize priming effects, a member of the research team will meet individually with each of the original stakeholders and present the cards (one statement per card) and ask each stakeholder to sort similar statements into the same pile, yielding as many piles as he/she deems appropriate [62]. Finally, each participant will be asked to rate each statement on a 1 to 5 point scale on “Importance” (from 1 – “Not at all important” to 5 – “Extremely important”) and “Changeability” (from 1 – “Not at all changeable” to 5 – “Extremely changeable”). This may appear a daunting task, but we have successfully used such techniques with mental health consumers in the past.

Multidimensional scaling and hierarchical cluster analysis will then be used to generate a visual display of how statements are clustered across all participants [63]. The result will be a single “Concept Map” depicting which statements participants had frequently sorted together. Multiple study investigators will independently evaluate potential solutions (e.g., 12 clusters, 15 clusters) and agree on the final model based on a statistical “stress” value and interpretability [62, 63]. CM requires small sample sizes and having 4–6 participants for each stakeholder group will suffice for this work [24]. Finally, study participants will reconvene with the research team in order to define the meaning and a name for each of the final clusters.

## Discussion

The bundling of CBT and SST into the CBSST intervention is an innovative and comprehensive approach to improving functioning in schizophrenia. Adapting CBSST to fit into the ACT service delivery context commonly found throughout the United States creates an opportunity to substantially increase the number of persons with schizophrenia who could have access to and benefit from CBT and SST interventions that target impaired functioning in consumers with schizophrenia. A demonstration of the effectiveness of ACT + CBSST and the identification of important implementation barriers and facilitators through this hybrid research study may result in greater availability of these interventions in this population. To our knowledge, this is the first effectiveness study of CBT and SST for schizophrenia implemented on ACT teams by frontline providers in the United States. Additionally, as a hybrid effectiveness and implementation study, we will identify barriers and facilitators to implementation of the CBSST program, enabling us to develop guidelines that will maximize successful implementation of the program in the future. Given this attention to implementation factors, we have been meeting with agency frontline staff and their supervisors as part of their ongoing training and to help troubleshoot challenges related to providing ACT + CBSST. This feedback has resulted in the adaptations described below.

### CBSST adaptations and lessons learned

First, it is imperative to promote buy-in and consistent support from agency leadership, both at the organizational level as well as the individual team level. Supervisors, in this case ACT team leaders, have been invaluable in assuring that CBSST is delivered consistently, that case managers communicate with each other about the treatment between sessions, and that specific times during team meetings are designated for discussing issues related to CBSST delivery. Supervisors have also been instrumental in assisting with logistics of the implementation, including providing case managers with recorders and forms for documenting and tracking session delivery, collecting necessary forms, and providing feedback to the consultants about the consultation meetings and the study overall. Ideally, supervisors also participate in the study and work towards certification in CBSST, so that they truly serve as a role model for all other team members. Materials that facilitate communication about CBSST delivery between case managers have proven extremely helpful, including a whiteboard set up in the team room which includes information about current study participants, their individual recovery goals, and the most recent session they have completed. Maintaining a “team” version of the manual that mirrors that of the consumer and can then be shared with the provider who will be delivering the subsequent session. Finally, ensuring that study materials such as recorders with extra batteries, workbooks, and forms, are readily available to team members and carried with providers on a daily basis, in some cases, seems to increase compliance with completing sessions.

At the provider level, anxieties about delivering an unfamiliar intervention and being recorded and evaluated should be openly elicited and addressed. Consultants continually normalize the experience of being recorded for fidelity and provide written and verbal feedback that balances specific and directive suggestions for improving fidelity with positive reinforcement of providers’ strengths and growth. It has been helpful for consultants to identify and directly address negative beliefs and myths associated with implementing manualized interventions, such as CBSST (e.g., that CBSST is too difficult or rigid, that it will not help clients in crisis, etc.). Providers have appreciated case examples and role plays illustrating how CBSST material can be integrated into a typical ACT session and how the treatment is tailored to challenging presentations (e.g., thought disorder, negative symptoms, entrenched delusions). In addition, providers have responded positively to simple incentives including food, certificates of high-fidelity CBSST delivery, and public recognition of exceptional sessions. In general, CBSST implementation tends to be optimal when the perception that the treatment does not substantially increase staff workload or reduces burden by reducing consumer crises, and provider confidence and efficacy is strongly supported.

### Improved training materials

Based upon feedback from the ACT providers, new training materials were developed to provide greater opportunities for observing and practicing using CBSST prior to delivering it to ACT consumers (e.g., online didactic videos and additional examples). Additionally, the CBSST workbooks were revised and abbreviated to promote ease of use for ACT providers and consumers in the community during typically brief visits when compared to a traditional office-based session. A primary goal for the revised training materials and approaches was to help ACT providers become sufficiently comfortable with the CBSST intervention so that they could naturally integrate it into their typical services and tailor it to helping consumers achieve their goals, as well as to better manage everyday and crisis situations. As providers attain competence in CBSST through practice, they begin to view these situations as good opportunities to utilize CBSST rather than as barriers to its service delivery.

## Trial status

Study recruitment, assessment, and intervention delivery activities are ongoing for the effectiveness component of the hybrid research study. Follow-up data collection is anticipated to be completed by June 2016. For the implementation study, we are in the process of collecting Concept Mapping “brainstorming” statements from multiple stakeholder groups related to the factors that have influenced ACT + CBSST implementation to date.

## Abbreviations

ACT: Assertive Community Treatment
BPRS: Expanded Brief Psychiatric Rating Scale
CBSST: Cognitive Behavioral Social Skills Training
CBT: cognitive behavior therapy
CMT: Comprehensive Modules Test
CPZE: chlorpromazine equivalents
CTS-Psy: Cognitive Therapy Scale for Psychosis
DAS: Dysfunctional Attitude Scale
DACTS: Dartmouth Assertive Community Treatment Scale
DPAS: Defeatist Performance Attitude Scale
DSM-IV-TR: *Diagnostic and Statistical Manual of Mental Disorders, 4th edition, text revision*
EBPs: evidence-based practices
GFSC: goal-focused supportive contact condition
ICC: intraclass correlation coefficient
ILSS: Independent Living Skills Survey
MASC: Maryland Assessment of Social Competence
NIMH: National Institute of Mental Health
SANS: Scale for the Assessment of Negative Symptoms
SCID: Structured Clinical Interview for DSM-IV
SST: social skills training
TAU: Treatment as Usual
UCSD: University of California, San Diego

## References

[1] Lehman AF, Steinwachs DM (1998). Patterns of usual care for schizophrenia: initial results from the Schizophrenia Patient Outcomes Research Team (PORT) client survey. Schizophr Bull.

[2] Curran GM, Bauer M, Mittman B, Pyne JM, Stetler C (2012). Effectiveness-implementation hybrid designs: combining elements of clinical effectiveness and implementation research to enhance public health impact. Med Care.

[3] Green MF (1996). What are the functional consequences of neurocognitive deficits in schizophrenia?. Am J Psychiatry.

[4] Green MF, Kern RS, Heaton RK (2004). Longitudinal studies of cognition and functional outcome in schizophrenia: implications for MATRICS. Schizophr Res.

[5] Green MF, Nuechterlein KH, Kern RS, Baade LE, Fenton WS, Gold JM (2008). Functional co-primary measures for clinical trials in schizophrenia: results from the MATRICS Psychometric and Standardization Study. Am J Psychiatry.

[6] Bowie CR, Reichenberg A, Patterson TL, Heaton RK, Harvey PD (2006). Determinants of real-world functional performance in schizophrenia subjects: correlations with cognition, functional capacity, and symptoms. Am J Psychiatry.

[7] Keefe RS, Poe M, Walker TM, Kang JW, Harvey PD (2006). The Schizophrenia Cognition Rating Scale: an interview-based assessment and its relationship to cognition, real-world functioning, and functional capacity. Am J Psychiatry.

[8] Twamley EW, Doshi RR, Nayak GV, Palmer BW, Golshan S, Heaton RK (2002). Generalized cognitive impairments, ability to perform everyday tasks, and level of independence in community living situations of older patients with psychosis. Am J Psychiatry.

[9] Murray CJL, Lopez AD (1997). Global mortality, disability, and the contribution of risk factors: global burden of disease study. Lancet.

[10] Wiersma D, Wanderling J, Dragomirecka E, Ganev K, Harrison G, an der Heiden W (2000). Social disability in schizophrenia: its development and prediction over 15 years in incidence cohorts in six European centres. Psychol Med.

[11] Robinson DG, Woerner MG, McMeniman M, Mendelowitz A, Bilder RM (2004). Symptomatic and functional recovery from a first episode of schizophrenia or schizoaffective disorder. Am J Psychiatry.

[12] Ho BC, Andreasen N, Flaum M (1997). Dependence on public financial support early in the course of schizophrenia. Psychiatr Serv.

[13] Grant PM, Beck AT (2009). Defeatist beliefs as a mediator of cognitive impairment, negative symptoms, and functioning in schizophrenia. Schizophr Bull.

[14] Wykes T, Steel C, Everitt B, Tarrier N (2008). Cognitive behavior therapy for schizophrenia: effect sizes, clinical models, and methodological rigor. Schizophr Bull.

[15] Benton MK, Schroeder HE (1990). Social skills training with schizophrenics: a meta-analytic evaluation. J Consult Clin Psychol.

[16] Kurtz MM, Mueser KT (2008). A meta-analysis of controlled research on social skills training for schizophrenia. J Consult Clin Psychol.

[17] Granholm E, McQuaid JR, McClure FS, Auslander LA, Perivoliotis D, Pedrelli P (2005). A randomized, controlled trial of cognitive behavioral social skills training for middle-aged and older outpatients with chronic schizophrenia. Am J Psychiatry.

[18] Granholm E, McQuaid JR, McClure FS, Link PC, Perivoliotis D, Gottlieb JD (2007). Randomized controlled trial of cognitive behavioral social skills training for older people with schizophrenia: 12-month follow-up. J Clin Psychiatry.

[19] Wallace CJ, Liberman RP, Tauber R, Wallace J (2000). The independent living skills survey: a comprehensive measure of the community functioning of severely and persistently mentally ill individuals. Schizophr Bull.

[20] Granholm E, Holden J, Link PC, McQuaid JR. Randomized clinical trial of cognitive behavioral social skills training for schizophrenia: improvement in functioning and experiential negative symptoms. J Consult Clin Psychol. 2014 10.1037/a0037098 PMC4244255 24911420

[21] Granholm E, Holden J, Link PC, McQuaid JR, Jeste DV (2013). Randomized controlled trial of cognitive behavioral social skills training for older consumers with schizophrenia: defeatist performance attitudes and functional outcome. Am J Geriatr Psychiatry.

[22] Drake RE, Bond GR, Essock SM (2009). Implementing evidence-based practices for people with schizophrenia. Schizophr Bull.

[23] Mueser KT, Bond GR, Drake RE, Resnick SG (1998). Models of community care for severe mental illness: a review of research on case management. Schizophr Bull.

[24] Trochim WMK (1989). An Introduction to concept mapping for planning and evaluation. Eval Program Plann.

[25] Granholm E, Loh C, Link PC, Jeste DV (2010). Feasibility of implementing cognitive behavioral therapy for psychosis on assertive community treatment teams: a controlled pilot study. Int J Cogn Ther..

[26] American Psychiatric Association. Diagnostic and statistical manual of mental disorders (DSM-IV). 4th ed. Washington, DC; 1994.

[27] Angermeyer MC, Kuhn L, Goldstein JM (1990). Gender and the course of schizophrenia - differences in treated outcomes. Schizophr Bull.

[28] Haas GL, Garratt LS, Mueser KT, Tarrier N (1998). Gender differences in social functioning. Handbook of social functioning in schizophrenia.

[29] Häfner H, an der Heiden W, Mueser KT, Jeste D (2008). Course and outcome. Clinical handbook of schizophrenia.

[30] Mueser KT, Bellack AS, Morrison RL, Wade JH (1990). Gender, social competence, and symptomatology in schizophrenia: a longitudinal analysis. J Abnorm Psychol.

[31] Mueser KT, Pratt SI, Bartels SJ, Forester B, Wolfe R, Cather C (2010). Neurocognition and social skill in older persons with schizophrenia and major mood disorders: an analysis of gender and diagnosis effects. J Neurolinguistics.

[32] Usall J, Haro JM, Ochoa S, Marquez M, Araya S (2002). Influence of gender on social outcome in schizophrenia. Acta Psychiatr Scand.

[33] Granholm E, McQuaid JR, McClure FS, Pedrelli P, Jeste DV (2002). A randomized controlled pilot study of cognitive behavioral social skills training for older patients with schizophrenia. Schizophr Res.

[34] McQuaid JR, Granholm E, McClure FS, Roepke S, Pedrelli P, Patterson TL (2000). Development of an integrated cognitive-behavioral and social skills training intervention for older patients with schizophrenia. J Psychother Prac Res.

[35] Beck AT, Rector NA (2000). Cognitive therapy of schizophrenia: a new therapy for the new millennium. Am J Psychother.

[36] Beck AT, Rush AJ, Shaw BF, Emery G (1979). Cognitive therapy of depression.

[37] Beck JS (1995). Cognitive therapy: basics and beyond.

[38] Kingdon DG, Turkington D (1994). Cognitive-behavioral therapy of schizophrenia.

[39] Liberman RP. Psychiatric rehabilitation consultants: modules in the UCLA social and independent living skill series. Camarillo: Psychiatric Rehabilitation Consultants; 1991. http://www.psychrehab.com.

[40] Bellack AS, Mueser KT, Gingerich S, Agresta J (2004). Social skills training for schizophrenia: a step-by-step guide.

[41] Thase ME, Kingdon D, Turkington D (2014). The promise of cognitive behavior therapy for treatment of severe mental disorders: a review of recent developments. World Psych.

[42] Teague GB, Bond GR, Drake RE (1998). Program fidelity in assertive community treatment: development and use of a measure. Am J Orthopsychiatry.

[43] Haddock G, Devane S, Bradshaw T, McGovern J, Tarrier N, Kinderman P (2001). An investigation into the psychometric properties of the Cognitive Therapy Scale for Psychosis (Cts-Psy). Behav Cogn Psychoth.

[44] Bellack AS, Sayers M, Mueser KT, Bennett M (1994). Evaluation of social problem solving in schizophrenia. J Abnorm Psychol.

[45] Sayers MD, Bellack AS, Wade JH, Bennett ME, Fong P (1995). An empirical method for assessing social problem solving in schizophrenia. Behav Modif.

[46] Cane DB, Olinger LJ, Gotlib IH, Kuiper NA (1986). Factor structure of the dysfunctional attitude scale in a student population. J Clin Psychol.

[47] Weissman AR (1978). The dysfunctional attitude scale: a validation study (doctoral dissertation, University of Pennsylvania, 1978). Diss Abstr Int..

[48] Andreasen NC (1982). Negative symptoms in schizophrenia. Definition and reliability. Arch Gen Psychiatry.

[49] Sayers SL, Curran PJ, Mueser KT (1996). Factor structure and construct validity of the scale for the assessment of negative symptoms. Psychol Assess.

[50] Lukoff D, Nuechterlein KH, Ventura J (1986). Manual for the expanded brief psychiatric rating scale. Schizophr Bull..

[51] Laird NM, Ware JH (1982). Random-effects models for longitudinal data. Biometrics.

[52] Hedeker D, Gibbons RD, Davis JM (1991). Random regression-models for multicenter clinical-trials data. Psychopharmacol Bull.

[53] Kraemer HC, Wilson GT, Fairburn CG, Agras WS (2002). Mediators and moderators of treatment effects in randomized clinical trials. Arch Gen Psychiatry.

[54] Diggle P, Liang K-Y, Zeger SL (1994). Analysis of longitudinal data. Oxford statistical science series, vol. 13.

[55] Hedeker D, Gibbons RD, Waternaux C (1999). Sample size estimation for longitudinal designs with attrition: comparing time-related contrasts between two groups. J Educ Behav Stat.

[56] Jensen PS (2003). Commentary: the next generation is overdue. J Am Acad Child Adolesc Psychiatry.

[57] Damschroder LJ, Aron DC, Keith RE, Kirsh SR, Alexander JA, Lowery JC (2009). Fostering implementation of health services research findings into practice: a consolidated framework for advancing implementation science. Implement Sci..

[58] Greenhalgh T, Robert G, Macfarlane F, Bate P, Kyriakidou O (2004). Diffusion of innovations in service organizations: systematic review and recommendations. Milbank Q.

[59] Trochim WM, Cabrera DA, Milstein B, Gallagher RS, Leischow SJ (2006). Practical challenges of systems thinking and modeling in public health. Am J Public Health.

[60] Trochim WM, Milstein B, Wood BJ, Jackson S, Pressler V (2003). Setting objectives for community and systems change: an application of concept mapping for planning a statewide health improvement initiative. Health Promot Pract.

[61] Aarons GA, Wells RS, Zagursky K, Fettes DL, Palinkas LA (2009). Implementing evidence-based practice in community mental health agencies: a multiple stakeholder analysis. Am J Public Health.

[62] Rosenberg S, Kim M (1975). The method of sorting as a data-gathering procedure in multivariate research. Multivariate Behav Res..

[63] Davison ML (1983). Multidimensional scaling.

